# Serum IgG4-negative IgG4-related disease with a cardiac mass: A case report

**DOI:** 10.1097/MD.0000000000034533

**Published:** 2023-08-04

**Authors:** Kensuke Namba, Daiki Sakai, Hiroshi Mikamo, Yuta Sugizaki, Yoshiya Sugiura, Nobuyuki Hiruta, Yasuo Matsuzawa, Kaichi Kaneko

**Affiliations:** a Division of Rheumatology, Department of Internal Medicine, Toho University Sakura Medical Center, Sakura, Japan; b Division of Respiratory Medicine, Department of Internal Medicine, Toho University Sakura Medical Center, Sakura, Japan; c Division of Cardiology, Department of Internal Medicine, Toho University Sakura Medical Center, Sakura, Japan; d Department of Surgical Pathology, Toho University Sakura Medical Center, Sakura, Japan.

**Keywords:** cardiac mass, IgG4-related disease, rituximab

## Abstract

Although IgG4-related disease (IgG4-RD) can affect various organs, its association with a cardiac mass is exceptionally rare. Here, we report a case of a woman with IgG4-RD and a cardiac mass and discuss 10 similar cases reported previously.

**Patient concerns:**

A 65-year-old woman was referred to our hospital for chest discomfort and back pain.

**Diagnoses:**

In accordance with the 2019 ACR/EULAR diagnostic criteria for IgG4-RD, she was diagnosed with IgG4-RD based on dense lymphocytic infiltration on histopathology, IgG/IgG4-positive cell ratio <40%, >10/hpf IgG4-positive cells on immunostaining, and paraspinal zone soft tissue lesions in the chest.

**Interventions:**

An external pacemaker was implanted for the complete atrioventricular block on the electrocardiogram. After the diagnosis of IgG4-RD, she was treated with glucocorticoids and rituximab.

**Outcomes:**

She remains under observation without disease recurrence.

**Lessons:**

IgG4-RD are usually treated with glucocorticoids; however, in cases of a cardiac mass, life-threatening complications may occur and surgery is often needed. Combination therapy with glucocorticoids and rituximab may be effective even in patients with IgG4-RD and cardiac mass, which may avoid the need of invasive treatments, such as surgery.

## 1. Introduction

IgG4-related disease (IgG4-RD) is a systemic fibroinflammatory disease characterized by tumefactive lesions, dense lymphoplasmacytic infiltrates rich in IgG4-positive plasma cells, storiform fibrosis, and, often, elevated serum IgG4 levels.^[1]^ Most patients are men and aged > 50 years.^[2,3]^ Glucocorticoids are the treatment of choice. Several disease-modifying antirheumatic drugs, such as methotrexate and azathioprine, have been used as steroid-sparing treatment of IgG4-RD, although studies confirming their efficacy are lacking.^[4]^ International consensus guidance statement suggest that patients should be treated initially with 0.6 kg/day of prednisolone for 2 to 4 weeks.^[4]^ In 2001, Hamano et al first reported that patients with sclerosing pancreatitis have high serum IgG4 levels and diffuse infiltration of IgG4-positive plasma cells in the pancreas; since then, abundant infiltration of IgG4-positive plasma cells has been demonstrated in many extra-pancreatic sclerosing lesions.^[5,6]^ IgG4-RD can affect almost all organ systems, but rarely affects the heart.^[1,7]^ We report a case of serum IgG4-negative IgG4-RD with a cardiac mass, which responded well to glucocorticoids and rituximab.

## 2. Case report

**Figure 1.**
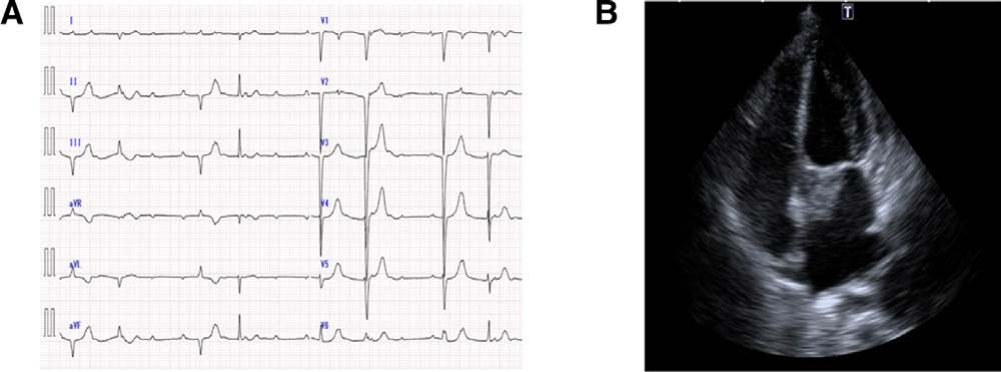
Complete atrioventricular block on electrocardiogram (A) and mass lesion on echocardiography (B).

**Figure 2.**
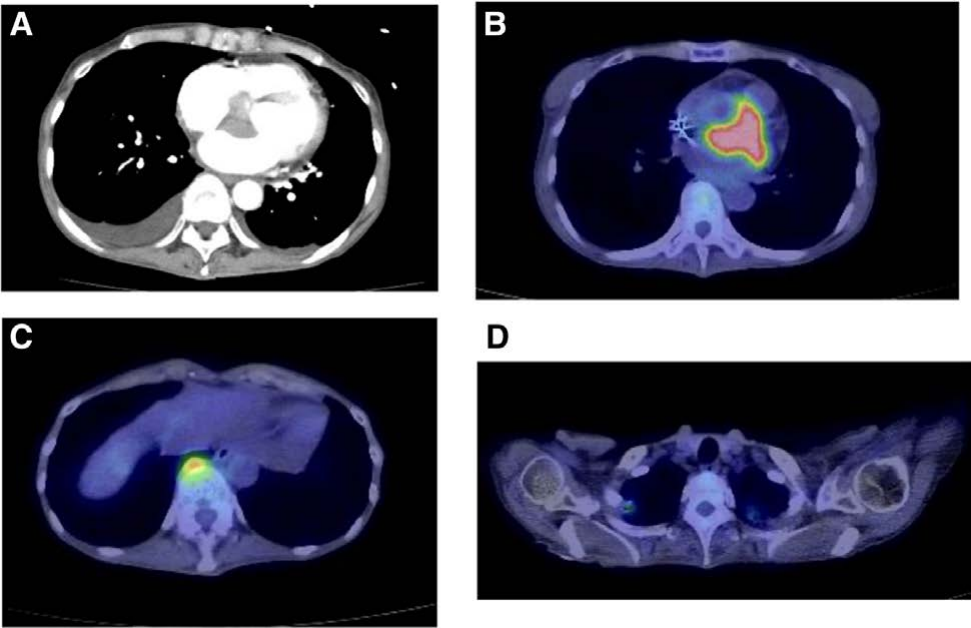
Mass lesion on CT (A). ^18^F-FDG-PET/CT showing uptake in the cardiac mass (B), anterior mass of the twelfth thoracic vertebra (C), and both lung apices (D).

**Figure 3.**
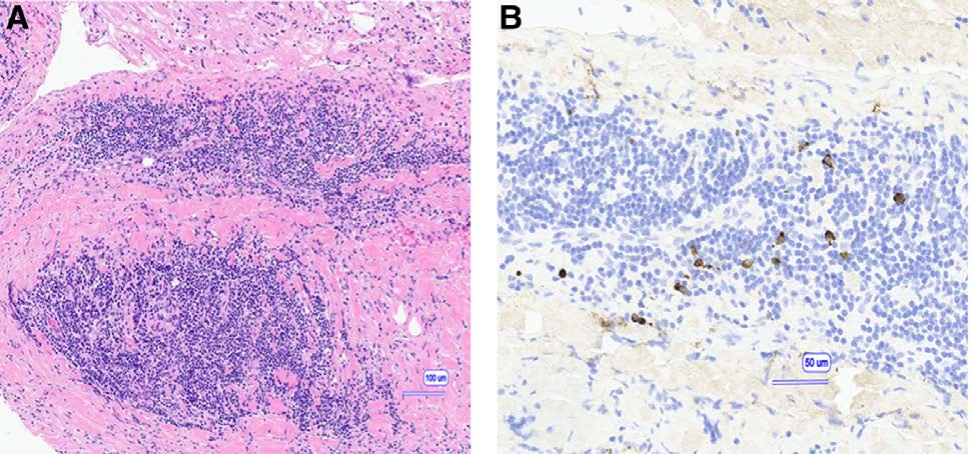
Microscopic section from the anterior mass of the twelfth thoracic vertebra with hematoxylin and eosin (A) and IgG4 (B) immunostaining. Original magnification: 100× (A), 200× (B).

**Figure 4.**
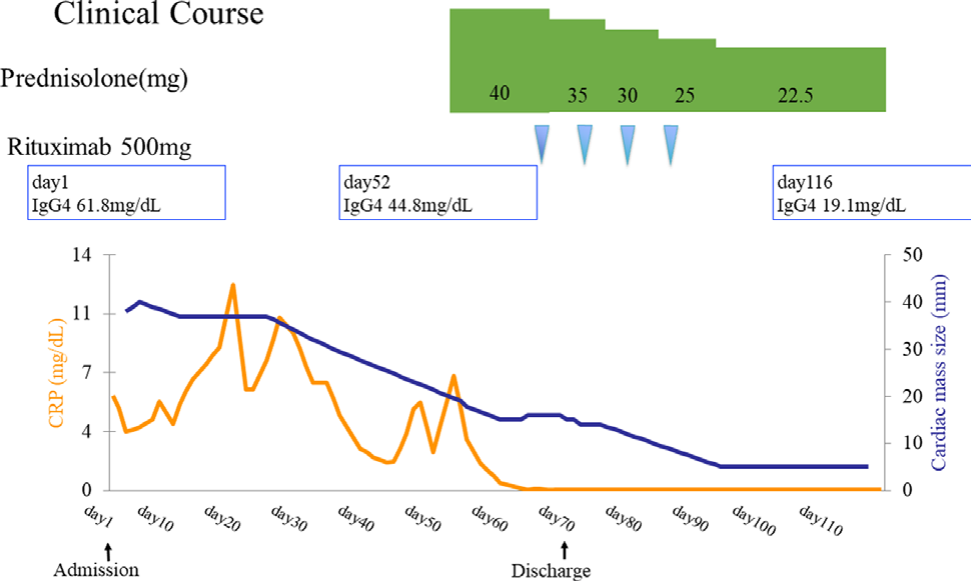
Clinical course of the patient.

A 65-year-old woman was referred to our hospital for chest discomfort and back pain. She had a history of iron-deficiency anemia. Upon physical examination at admission, she had systolic murmurs in the aortic valve region on cardiac auscultation. The electrocardiogram performed at admission showed complete atrioventricular block (Fig. 1A), for which an external pacemaker was implanted. Table 1 presents the laboratory data. The blood cell counts and liver and renal function tests were normal. The C-reactive protein (CRP) level was 5.67 mg/dL (normal range: 0–0.3) and the erythrocyte sedimentation rate was 40 mm/h (normal range: 0–20), indicating an inflammatory response. The serum brain natriuretic peptide level was 323.5 pg/mL (normal range: 0–18.4). Serum IgG and IgG4 levels were 1319 mg/dL (normal range: 870–1700) and 61.8 mg/dL (normal range: 4–108), respectively, whereas the other immunoglobulin levels were normal. The antinuclear antibody titer was <40-fold of normal. Echocardiography and contrast-enhanced computed tomography (CT) showed a 38 × 24-mm mass extending from the aortic valve to the left atrium (Figs. 1B and 2A). Considering the possibility of valve vegetations, blood cultures were performed 4 times, but there was no bacterial growth. ^18^F-fluorodeoxyglucose positron emission tomography/CT showed uptake in the cardiac mass (maximum standardize uptake value [SUVmax] = 10.6), anterior mass of the twelfth thoracic vertebra (SUVmax = 4.5), and both lung apices (SUVmax = 2.6) (Fig. 2B–D). Biopsy of the cardiac mass was not performed because of the risk of distant metastasis if the mass was a cardiac sarcoma. A biopsy of the mass in the anterior aspect of the twelfth thoracic vertebra was performed. At a high magnification level, fibrosis in the fat, lymphoplasmacytic infiltration, and fibrous bundles were observed (Fig. 3A). IgG4-RD was suspected, and immunostaining was performed. Although the average IgG4/IgG ratio was <40%, >10 IgG4-positive cells per field of view were observed at a high magnification at the hot spot (Fig. 3B). Based on the dense lymphocytic infiltration on histopathological analysis, IgG/IgG4-positive cell ratio <40%, >10/hpf IgG4-positive cells on immunostaining, and paraspinal zone soft tissue lesions in the chest, she was diagnosed with IgG4-RD in accordance with the 2019 ACR/EULAR IgG4-RD diagnostic criteria.^[8]^ As shown in Figure 4, the patient was treated with 40 mg of prednisolone (1 mg/kg/d) from day 52 of hospitalization. Subsequently, the cardiac mass and the anterior mass of the twelfth thoracic vertebra shrank; furthermore, the serum IgG4 and CRP levels decreased. She had a residual cardiac mass, and was treated with 4 days of 500 mg/wk of rituximab from day 69 of hospitalization. Thereafter, the cardiac mass further decreased in size and the CRP level normalized. Prednisolone was tapered to 30 mg/d on day 73 of hospitalization and she was discharged on day 78.

**Table 1:** Laboratory findings at admission.

WBC	8720/μL	CRP	5.67 mg/dL	IgG	1319 mg/dL
Neu	67.3%	TP	7.2 g/dL	IgA	257 mg/dL
Eos	0.6%	Alb	2.8 g/dL	IgM	68 mg/dL
Ba	0.2%	AST	20 IU/L	IgG4	61.8 mg/dL
Mo	6.4%	ALT	6 IU/L	CH50	49.7 U/mL
Ly	25.5%	LDH	271 IU/L	C3	124 mg/dL
RBC	478 × 104/μL	T-Bil	<0.1 mg/dL	C4	27 mg/dL
MCV	81fl	Cr	0.51 mg/dL	sIL2-R	525 U/mL
MCH	24.3 pg	Na	138 mEq/L	ANA	< 40 times
MCHC	30.0 g/dL	K	4.4 mEq/L	PT	83 %
Hb	11.6 g/dL	Cl	103 mEq/L	APTT	22.3 s
Ht	38.7%	Ca	8.7 mg/dL	D-dimer	1.54 μg/mL
Plt	41.4/μL	BNP	323.5 pg/mL		
ESR	40 mm/h	Ferritin	218.14 ng/mL		

## 3. Discussion

IgG4-RD was initially identified in patients with autoimmune pancreatitis and an elevated serum IgG4 level. It is characterized by extensive infiltration of IgG4-positive plasma cells and T lymphocyte in various organs and storiform fibrosis. IgG4-RD rarely affects the heart. To the best of our knowledge, there have been 10 previous reports on IgG4-RD affecting the heart (Table 2).^[7,9–17]^ Among these cases, the mean patient age was 62 years. In a previous study of 493 patients, the mean ages at symptom onset for patients from Asian and non-Asian countries were 57.7 and 59.5 years, respectively.^[18]^ The location of the cardiac masses was variable; however, the salivary glands were the most common site of extracardiac lesions. Serum IgG4 levels were measured in 6 patients, with a mean value of 800 mg/dL. The pretreatment serum IgG4 level was normal in only a single patient. In a cross-sectional study, serum IgG4 levels were elevated in 86% of 114 patients.^[19]^ Among patients with IgG4-RD and a cardiac mass, the proportion of patients with normal serum IgG4 level was similar to that among patients with typical IgG4-RD. IgG4-RD is usually treated with glucocorticoids; however, the association of IgG4-RD with a cardiac mass may lead to life-threatening complications and surgery is often required. Nevertheless, some such patients have been treated with only internal therapy. Matsumura et al reported a case in which the cardiac mass decreased after administration of 0.6 mg/kg/d of prednisolone, and the patient remained stable for 12 months.^[8]^ Carbajal et al reported a case in which the cardiac mass decreased in size in response to treatment with high-dose steroids.^[12]^ The combination of rituximab and glucocorticoids is particularly useful in patients who have steroid-resistant disease or are unsuitable for steroid use. Carruthers performed an open-label study of 30 patients with IgG4-RD who were treated with rituximab, most without glucocorticoids; 97% of patients had improved disease, and 40% maintained complete disease remission after 12 months.^[20]^ In our patient, rituximab was added to prednisolone when the latter was ineffective; the cardiac mass decreased in size in response to rituximab and prednisolone treatment. Our case suggests that combination therapy with glucocorticoids and rituximab may be effective even in patients with IgG4-RD and cardiac mass, which may avoid the need of invasive treatments, such as surgery.

**Table 2:** Cases of IgG4-related disease with a cardiac mass.

Case	Age	Sex	Serum IgG4 level (mg/dL)	Disease site	Treatment	Reference no.
1	63	F	456	CA and AAA	Surgery	^[9]^
2	75	M	2510	CA, pancreas, and parotid glands	Surgery and glucocorticoids	^[10]^
3	59	F	Not reported	SN, LV, parotid glands, and LN	Glucocorticoids	^[11]^
4	55	F	Not reported	RA and AS	Pacemaker	^[12]^
5	59	F	65.9 (postoperative)	LA	Surgery and pacemaker	^[13]^
6	58	F	64.2	PV	Surgery	^[14]^
7	64	F	Not reported	MV and AV	Surgery	^[15]^
8	52	M	Not reported	RV and TAA	Surgery and glucocorticoids	^[16]^
9	64	M	259	PV and RV	Surgery and glucocorticoids	^[17]^
10	69	M	1450	RA, AS, and lacrimal, salivary, and submandibular glands	Glucocorticoids	^[7]^
Present case	65	F	61.8	RA, AV, twelfth thoracic vertebra, and lung apices	Pacemaker, glucocorticoids, and rituximab	

AAA = abdominal aortic aneurysm, AS = atrial septum, AV = aortic valve, CA = coronary artery, F = female, LA = left atrium, LN = lymph node, LV = left ventricle, M = male, MV = mitral valve, PV = pulmonary valve, RA = right atrium, RV = right ventricle, SN = sinus node, TAA = thoracic aortic aneurysm.

## Author contributions

**Conceptualization:** Kaichi Kaneko.

**Writing – original draft:** Kensuke Namba, Kaichi Kaneko.

**Writing – review & editing:** Daiki Sakai, Hiroshi Mikamo, Yuta Sugizaki, Yoshiya Sugiura, Nobuyuki Hiruta, Yasuo Matsuzawa.
